# Unraveling the Immune Microenvironment of Thymic Epithelial Tumors: Implications for Autoimmunity and Treatment

**DOI:** 10.3390/ijms23147864

**Published:** 2022-07-16

**Authors:** Christos Masaoutis, Kostas Palamaris, Stefania Kokkali, Georgia Levidou, Stamatios Theocharis

**Affiliations:** 1 First Department of Pathology, Medical School, National and Kapodistrian University of Athens, 75, M. Asias Str., Bld 10, Goudi, GR11527 Athens, Greece; cmasaout@med.uoa.gr (C.M.); kpalamaris@yahoo.gr (K.P.); georgia.levidou@klinikum-nuernberg.de (G.L.); 2 Oncology Unit, 2nd Department of Medicine, Medical School, National and Kapodistrian University of Athens, Hippocratio General Hospital of Athens, 114, V. Sofias Str., GR11527 Athens, Greece; stefkokka@med.uoa.gr; 3 Second Department of Pathology, Paracelsus Medical University, 90419 Nurenberg, Germany

**Keywords:** TETs, thymoma, thymic carcinoma, tumor microenvironment, thymocytes, B-cells, immune checkpoint inhibitors, PDL-1, autoimmunity, myasthenia gravis

## Abstract

Thymic Epithelial Tumors (TETs) represent a rare tumor family, originating from the epithelial component of the thymus gland. Clinicopathologically, they are segregated into six major subtypes, associated with distinct histological features and clinical outcomes. Their emergence and evolution are accompanied by the generation of a complex tumor microenvironment (TME), dominated by phenotypically and functionally divergent immune cellular subsets, in different maturation states and in analogies that vary significantly among different subtypes. These heterogenous leukocyte populations exert either immune-permissive and tumor-suppressive functions or vice versa, and the dynamic equilibrium established among them either dictates the tumor immune milieu towards an immune-tolerance state or enables the development of a productive spontaneous tumoricidal response. The immunologically “hot” microenvironment, defining a significant proportion of TETs, makes them a promising candidate for the implementation of immune checkpoint inhibitors (ICIs). A number of phase I and II clinical trials have already demonstrated significant, type-specific clinical efficacy of PD-L1 inhibitors, even though substantial limitations in their utilization derive from their immune-mediated adverse effects. Moreover, the completed clinical studies involved relatively restricted patient samples and an expansion in the enrolled cohorts is required, so that more trustworthy conclusions regarding the benefit from ICIs in TETs can be extracted.

## 1. Introduction

Thymus malignancies represent an extremely rare and heterogenous tumor family that confines multiple different entities of heterologous origins [1]. The most prominent thymic neoplasms derive from the epithelial component of the gland (thymic epithelial tumors: TETs) and are classified into two broad categories: thymomas and thymic carcinomas (TCs). Thymomas and TCs are associated with perceived variations in their architectural patterns and cellular features, as well as in their biological behavior. From a histological perspective, TETs are composed of a combination of neoplastic thymic epithelial cells and non-neoplastic lymphocytes, admixed in various proportions. The latest WHO edition established a more analytical nomenclature scheme for the further classification of TETs, based predominantly on their differentiation towards cortical or medullary thymic epithelium, as well as their exact ratio differences in the epithelial and lymphocytic component. The different subtypes are also associated with distinguishing differences in their malignant potential, metastatic capability and prognosis. Thus, TETs are subdivided into six categories: class A, AB, B1, B2, B3 and C (WHO 2021, 5th edition) [2,3]. Tumors of A subtypes are considered as neoplasms of a medullary phenotype, while all B subtypes originate from the thymic cortex. AB tumors are regarded as hybrids, containing merged areas of both phenotypes. The first four subtypes (A, AB, B1, B2) are members of thymomas and they are tumors of better differentiation and more benign biological properties. Class C essentially refers to TC and accounts for malignant aggressive neoplasms, usually of squamous differentiation, which demonstrate enhanced invasive and metastatic capacity. The B3 subtype, also termed as atypical thymoma or well-differentiated thymic carcinoma, represents an entity with an intermediate clinicopathological profile and biological characteristics, between the more benign thymomas and class C carcinomas [2,3]. The therapeutic approach for TETs is based predominantly on surgical resection, while platinum-based chemotherapy is the treatment of choice in advanced-stage tumors [4].

Tumors are not isolated masses of proliferative neoplastic cells. Conversely, they should be regarded as complex ecosystems, composed of malignant cells surrounded by a dynamic microenvironment (tumor microenvironment: TME). Through a delicate network of bi-directional, non-linear interactions, the different compartments of TME directly shape tumor evolutionary course and modify intratumor phenotypic heterogeneity, by modulating the successive cycles of clonal emergence and selection pressure.

In this review, we summarize the accumulated knowledge concerning different components of the tumor immune microenvironment (TIME) and their roles in TETs, as well as immunologically crucial molecules, with potential clinical implications as therapeutic targets. We also analyze existing data regarding clinical studies aiming to evaluate the implementation of immunotherapeutic agents as a first-line treatment option in thymic neoplasms.

## 2. Tumor Immune Microenvironment

From the initial steps of malignant transformation, tumor cells serve as active orchestrators of a stroma remodeling process that stimulates a local-fibroinflammatory reaction. Their secretome is highly rich in paracrine soluble molecules, such as cytokines and growth factors, with the capability to recruit heterogenous mesenchymal and immune cell populations and evoke a reprogramming of their phenotypic and functional state, so that they can favor the generation of tumor-permissive and immune-suppressive conditions [5]. The major cellular constituents accrued at the TME are of divergent origin and engulf adipocytes, vascular endothelial cells, fibroblasts, pericytes and, chiefly, immune cells. These heterologous cell lineages exhibit a functional diversification and specialization [6]. Fibroblasts infiltrating TME are termed as cancer-associated fibroblasts (CAFs) and their functional significance in the context of malignancy emulates the role of their normal counterparts, as synthetic machines that secrete and deposit immense quantities of extracellular matrix (ECM) [7]. Endothelial cells formulate a de novo delicate vascular network that covers the continuously increasing demand of tumor cells for oxygen and nutrients [8]. The preponderant cellular subsets within TME are different leucocyte populations, of both myeloid and lymphocytic provenance. Since some immune cells exert tumor-restraining functions while others act in a tumor-permissive manner, it is plausible to speculate that the development of tumor-promoting inflammation and an anti-tumor immune response occur in parallel and establish conditions of a dynamic equilibrium within the TME. The affluence and activation state of different cell populations determine in which direction this delicate balance is tilted.

The main cellular representatives of tumor-promoting inflammation originate from a perturbation of bone marrow myeloid compartment. Tumor-associated macrophages (TAMs) and myeloid-derived suppressor cells (MDSCs) are innate immunity cells of myeloid origin, with a multipronged role in finely tuning tumor cell functions and suppressing effective priming of adaptive immunity cellular components [9,10]. On the other hand, the generation of a productive immune reaction depends on the activation of dendritic cells/CD8 cytotoxic T-cells axis [11]. Dendritic cells are the most conventional antigen-presenting cells and represent the bridge between innate and adaptive immunity. They have an enhanced capacity of uptaking, processing and presenting tumor-derived antigens. They are also equipped with scavenger receptors, able to sense damage-associated molecular patterns (DAMPs), released by dying transformed cells, thus, upregulating co-stimulatory molecules [11,12,13]. These two distinct signals enhance their full activation and differentiation, rendering them capable of inducing an effective priming of antigen-specific cytotoxic T-cells.

Taken together, components of TIME have different roles in modulating the promotion and progression of tumor and anti-tumor immune response.

## 3. Immune Cell Populations of Thymoma and Thymic Carcinoma

### 3.1. Adaptive Immunity

**Figure 1:**
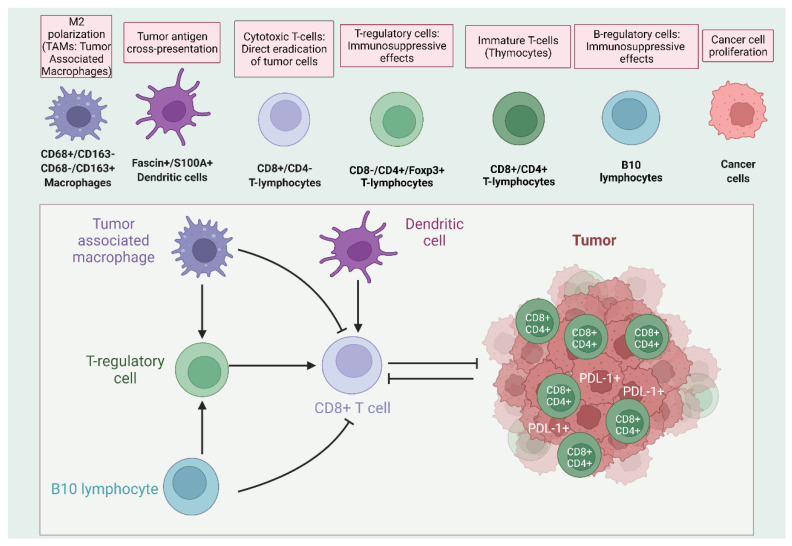
Tumor microenvironment of TETs contains multiple mature cell populations of innate and adaptive immunity, along with immature, double-positive (CD4+/CD8+) thymocytes. A complex network of interactions among these heterogenous cellular subsets shapes anti-tumor immune response and determines patient vulnerability for the development of autoimmune diseases.

TETs’ immune microenvironment is composed of multiple innate and adaptive immune cell populations with different phenotypes and heterogenous distribution within the tumor mass (Figure 1). T-cells, in different maturation and functional states, represent the most affluent cellular subset encountered in thymic neoplasms. In A/AB/B1/B2 thymomas, the majority of T-cell infiltration consists foremost of immature T-lymphocytes, with a double-positive (CD4+ CD8+) immunophenotypic profile, resembling that of normal thymocytes [14,15]. Thus, thymomas’ microenvironment, in large part, mimics the architecture of the normal thymus gland. On the contrary, T-cells recruited to B3 thymomas and thymic carcinomas are dominated by terminally differentiated CD4-only- and CD8-only-expressing lymphocytes. While T-cells are the effector compartment of cellular adaptive immunity, B-cells represent the executors of humoral immune responses. However, while their functions have been thoroughly examined in the context of autoimmune diseases [16], their contribution in tumor immunity has only recently begun to be investigated and elucidated [17,18,19]. In TETs, B-cells display a type-specific distribution. They are more affluent in a minority of tumors, termed as micronodular thymic neoplasms with follicular lymphoid hyperplasia that include micronodular thymoma with lymphoid B-cell hyperplasia, and micronodular carcinoma with lymphoid hyperplasia, where they form lymphoid aggregates with germinal centers [20,21]. Moreover, an immune infiltrate rich in CD20-positive B lymphocytes occurs in patients bearing, simultaneously, thymomas and autoimmune disorders, especially Myasthenia Gravis (MG). They are allocated in medullary islands of types A and B1, and form stromal aggregates in types B2 and B3 Thymomas [22,23].

### 3.2. Innate Immunity

Dendritic cells, as the professional antigen-presenting cell population in the immune system, orchestrate the effective priming of CD8 T-cells and produce soluble paracrine factors that recruit T-cells within TME, reinforcing local cytotoxic function (Figure 1) [24]. Thus, their phenotypic and functional integrity is a prerequisite for the occurrence of a de novo antigen-specific adaptive immune reaction. In TETs, mature dendritic cells’ immunophenotypic profile is characterized by Fascin and S100A expression, along with the absence of classic dendritic cells marker CD1a [25,26]. Immunohistochemical evaluation in resected thymus samples has revealed higher densities of such double-positive DCs in lower malignancy TETs subtypes, such as B1 and B2, while TCs are characterized by a scarcity of differentiated DCs [25,26].

In addition to dendritic cells, which serve as cellular intercessors between tumor cells and adaptive immunity, another prominent myeloid lineage population within TME, namely TAMs, carries out a converse function, by suppressing immune response and directly reinforcing tumor progression (Figure 1) [27,28,29]. Based on the expression profile of established macrophage markers, two distinct macrophage populations are characterized within TETs TME: one expressing CD68 and one expressing CD163 [26]. In general, higher levels of CD163+ TAMs compared to CD68+ are detected across all TET subtypes, while CD163+ population also exhibits a progressive enrichment along with the aggravation of tumor malignancy. Thus, TCs display the highest abundance of CD163+ compared to all thymoma types. On the other hand, there is no notable difference in the concentration of CD68+ macrophages among thymoma subtypes and TCs. Despite their distribution variations among different TET categories, both TAM populations (CD68+ and CD 163+) are associated with tumor invasion and nodular metastases [26,30].

## 4. Immune Targets and Immunotherapy in Thymoma and Thymic Carcinoma 

The tumor microenvironment (TME) of thymic neoplasms is of profound importance, from both a basic science and clinical perspective. The affluence and maturation state of different effector components of adaptive immunity is the decisive factor for the effective utilization of immunotherapeutic regimens, while it is also the main determinant for the occurrence of autoimmune conditions, related to TETs, such as MG. Moreover, as immune checkpoint inhibitors exploit the critical role of PD-L1/PD-1 and CTLA-4/CD80/CD86 axes in the evasion of immune surveillance, it is a normal consequence that the expression of PD-L1 on tumor cells is a sine qua non for their clinical efficacy [31]. Thus, the discrete composition of TME within different TET histological categories, along with the variabilities encountered among them in the expression patterns of PD-L1, constitute an integral part of their biological background and directly regulate both their response to checkpoint-inhibitory receptor blockades and to the autoimmunity predisposition [32]. 

Tumors of B1 and B2 classes, which are more frequently associated with autoimmunity, especially MG development, are characterized by an infiltrate dominated by two cellular subsets: immature T-cells, resembling normal thymocytes, and B-lymphocytes. This composition means that a spontaneous immune response against neoplastic cell fails to emerge, while it also provides a possible mechanistic link between the occurrence of such neoplasms and an autoimmunity predisposition. The immature state of double-positive lymphocytes renders them functionally defective and unable to elicit a productive tumoricidal immune response against tumor cells [33]. However, the T-cell population consists of numerous different clones, with a diverse array of antigenic specificities, including self-reactive lymphocytes, which represent latent sources of autoimmune reactions. Even though neoplastic cortical thymic epithelial cells retain some of their normal counterpart properties, such as the antigen presentation potential and the ability to induce anergy and deletion of self-reactive thymocytes, it seems that this process is severely compromised in the context of thymic tumorigenesis [33]. The most rigorous established theory, attempting to provide a mechanistic link between thymic epithelial neoplasms and autoimmunity susceptibility, is based on a disruption of thymocyte circulation between different compartments in the thymic parenchyma. This “escape” theory supports the notion that tumor-associated thymocytes fail to interact with medullary thymic epithelial and dendritic cells, which represent the key drivers in immune tolerance induction. Henceforth, self-reactive thymoma-derived thymocytes may escape into the circulation, avoiding critical medullary negative selection [33]. Regarding lymphocytes of the B-lineage, they also engulf populations with different phenotypic and functional properties. In tumor background, including TETs, the most abundant B-cell population displays a regulatory phenotype, termed as B10 cells, due to the secretion of immunosuppressive cytokine interleukin-10 (IL-10). Indeed, immunohistochemical analysis of epithelial thymic neoplasms, especially those associated with MG, revealed that tumor progression is accompanied by a gradual accumulation of B10 cells [34,35]. The latter, through secreting immense quantities of IL-10, as well as other immunosuppressive mediators, such as TGF-b, dampens the generation of a Th1-mediated adaptive immune response and enables the generation of large T-reg populations. They also circumvent CD8 T-cells and NK-cell-mediated cytotoxicity of tumor cells [36,37,38]. 

In contrast to the domination of immature T-cells and B10 in B1 and B2 tumor subtypes, in more aggressive TETs, the immune infiltrate is composed mainly of fully differentiated CD4 and CD8 single-positive T-lymphocytes, which represent the main cellular components of anti-tumor immune reactions. Specifically, CD8 cytotoxic cells act as “killing” machines and eliminate tumor cells, aiming to contain their outgrowth. However, while the presence of mature cytotoxic lymphocytes is a pre-requisite for an effective anti-tumor immune response, it is not itself a sufficient condition. Within TME, a large proportion of cytotoxic T-cells exhibits an exhausted phenotype, defined by the expression of immune checkpoint molecules, especially PD-1 and Tim3 and, as a consequence, their functional integrity is severely compromised and their tumor-restraining capability is abrogated [39]. Moreover, the recruitment of regulatory T-cells further contributes to the establishment of an immunosuppressive milieu within TME, as this CD4+/Foxp3+ mature subpopulation impairs the effective priming of CD8 T-cells [40]. Thus, while in B3 and C tumors a driving force for immune-mediated tumor elimination does exist, it might be severely compromised as a result of the contextual circumstances that undermine their functional integrity. 

The differences in the cellular populations encountered in different TET categories are also accompanied by distinct profiles regarding PD-L1/PD-1 expression levels and distribution within TIME. Numerous studies conducted with the aim of unraveling PD-L1 expression pattern in TETs notified higher levels of PD-L1 on TC and B3 thymomas compared to A, AB and B1 [41,42,43,44,45,46,47]. Besides the type-specific variations at the levels of PD-L1, additional factors seem to control its expression. More specifically, differences in its expression have been observed between primary and metastatic or recurrent TETs and between metastatic/recurrent tumors at different time points [48], while it has also been shown that chemotherapy substantially increases PD-1/PD-L1 levels. The later finding, of association between chemotherapy administration and increased PD-L1 expression, presumes that sequential therapeutic protocols, based on initial administration of chemotherapeutic factors, preceded by immune checkpoint inhibitors (ICIs), could considerably propagate immunotherapy tumor-suppressing capacity [49]. As the method of immunohistochemical assessment of PD-L1 levels has been disputed and the differences encountered in multiple studies are partly believed to stem from differences in antibodies and scoring methods implemented in staining evaluation [43,50], a number of studies focused on the evaluation of PD-L1 gene transcript levels. Consistent with antibody-mediated methods, disease progression to more advanced stages was accompanied by a gradual increase in PD-L1 transcriptional rate, while more aggressive TETs of B2 and B3 subtypes were also related to higher PD-L1 mRNA levels [51]. 

As the expression of PD-L1 in different subtypes of thymic neoplasm has begun to be elucidated, recent efforts have focused on mechanisms that control its expression patterns, which seems to be, at least partly, affected by the heterogeneity of TME. Immunohistochemical analysis of TET surgical specimens indicated that TME of highly aggressive B2 and B3 TETs is defined by an enrichment in anti-inflammatory cytokine IL-10, which exerts pleotropic inhibitory effects on different populations of T-cell lineage and mediates the circumvention of adaptive immunity. In addition to its integrative role in promoting immunosuppression, it is also associated with increased levels of PDL-1 [51].

## 5. Predictors of TET Response to Immunotherapy

Due to the progressively growing application of PD-L1 inhibitors in malignancy treatment, extensive research efforts have been devoted in the pursuit to identify promising biomarkers, which could predict the potential benefit from immune modulation in different patients. Besides PD-L1 expression in tumor cells [52], several studies from a variety of neoplasms indicate a trend towards better response in cases with high tumor mutational burden (TMB) or those characterized by a dense CD8 T-cell lymphocytic infiltrate within the tumor microenvironment. High mutational burden is associated with larger quantities of neo-antigens, susceptible to recognition by cellular adaptive immunity, while spontaneous recruitment of cytotoxic T-cells creates the required driving force for the eradication of neo-antigen-expressing tumor cells, upon PDL-1 blockade [53,54]. In the setting of TETs, high TMB is rarely encountered and it is almost exclusively restricted to cases occurring in a Lynch syndrome background [55]. Hence, microsatellite instability (MSI) evaluation could prove a useful strategy, in order to predict the potential efficacy of PD-L1 inhibition. Such a strategy has already begun to be deployed in a wide range of malignant tumors related to MSI. Regarding infiltration of TME by cytotoxic T-cells and the generation of an immunologically “hot” microenvironment, there seems to be a type-specific composition of immune cell infiltrates among different TET subclasses, as already addressed in the previous section. Hence, the increased populations of single-positive CD4 and CD8 T-cells, encountered in B2/B3 thymomas and thymic carcinomas, predispose to higher sensitivity to PD-L1 inhibition and render these TET subtypes more vulnerable to elimination by ICIs. A clinical study of the commonly utilized anti-PD-L1 molecule avelumab was accompanied by tumor gene expression analysis and immune cell profiling in the peripheral blood of patients. Results demonstrated a correlation for a T-cell-inflamed IFNγ-related signature, increased lymphocyte levels, reduced B-cells and elevated T-cell repertoire diversity with improved patient response to immunotherapy [56,57].

## 6. Clinical Trials of PD-L1/PD1 Inhibitors in TETs

After some initial testing of PD-L1 inhibitors in isolated cases of TETs that showcased clinical efficacy, ICIs prevailed as a promising therapeutic approach in the effort to combat this rare and heterogenous tumor family [58,59]. As a result, a number of phase I and II clinical trials were conducted, in order to evaluate the safety and the potential benefit of administering PDL-1 inhibitors in TETs patients (Table 1). The three ICIs, which have so far been tested in a clinical setting, are pembrolizumab, nivolumab and avelumab. A phase I study implemented avelumab monotherapy in eight patients with TETs. In seven cases, PD-L1 inhibition demonstrated some clinical benefit: four patients exhibited partial response, while three experienced disease stabilization. Another phase I clinical trial evaluated avelumab efficacy in patients bearing a wide range of locally advanced metastatic solid malignancies, including eight TET cases, refractory to previous therapeutic protocols. Three of the eight patients with TETs experienced partial response. Similar results were extracted from a phase I study, implementing nivolumab in patients with solid tumors. One of the two patients with thymic carcinoma exhibited partial response. In general, the drugs administered in the above studies were well-tolerated. However, a number of severe (Grade 3 and 4) immune-related adverse effects occurred in a few cases but regressed after corticosteroid treatment [57,60,61]. 

A number of phase II clinical trials evaluating the potential clinical benefit from pembrolizumab treatment in thymic carcinoma have been completed or are currently in progress [62,63,64]. Giaccone et al. investigated 23 thymic carcinoma patients without other malignancy or autoimmune disease, after at least one line of chemotherapy. Disease progression was assessed and a response rate of 24% was detected; one patient achieved complete response, four achieved partial response, none achieved stable disease and seven had disease progression. In general, toxicity was well-tolerated, with fever, mild fatigue, diarrhea and rhinorrhea being the main adverse effects. However, one patient developed myocarditis requiring pace-maker implantation and in two cases, serious autoimmune disorders emerged after treatment discontinuation. 

Cho et al. tested pembrolizumab efficacy in 33 TET patients (7 with thymomas and 26 with thymic carcinomas). The eligibility criteria included progression after platinum-based chemotherapy and excluded the patients with autoimmune diseases [57]. The overall response rate was 24.2%; 8 patients reported partial response, 17 had stable disease and 8 had progressive disease. This study reported various ≥ grade 3 immunological side effects, including myasthenia gravis, hepatitis, thyroiditis, glomerulonephritis, subcutaneous myoclonus and colitis. 

These studies suggested that responses against TETs can be induced by pembrolizumab in different clinical settings. However, it also prompts that extensive surveillance is required for designing future studies, particularly focused on immunological adverse events. Various autoimmune disorders, mainly myasthenia gravis, complicate thymoma; the underlying pathology of these disorders is not known completely and may be associated with the combination of immune tolerance mitigation incited by thymic neoplasms and a disruption in the immune-suppression axis, evoked by PD-1/PD-L1 inhibition.

Henceforth, extensive discussions by multidisciplinary experts are required for the off-label use of PD-1/PD-L1 blockade agents for TETs. Clinical trials that are in progress for checkpoint inhibitors in TETs are enlisted in Table 1.

## 7. Discussion

TETs encircle multiple histological entities, associated with distinct clinical outcomes and responses to treatment. The heterogeneity of TME is an integral part of TET pathological diversity and it seems to be directly associated with both their differentiation and their molecular background. Thus, B subtype tumors represent neoplastic analogues of the thymus cortex, with progressive exacerbation of their biological aggressiveness and malignant traits. B1 tumors very closely resemble thymus cortical region, dominated by an ample population of immature, antigen-naïve thymocytes, admixed with scattered neoplastic thymic epithelial cells. Higher-grade tumors are defined by a progressive constriction of thymocyte population and an increase in tumor epithelial component. In B3 thymomas, as well as in thymic carcinomas, an immunologically “hot” microenvironment, with a lymphocytic infiltrate, dominated by mature CD8 T-cells, which exhibit an exhausted phenotype, is usually encountered. Based on the diversity of cellular composition observed among different TET subtypes, we could identify two distinct immune cell signatures with direct clinical implications. The first is detected in B1 and B2 tumors and it is characterized by an abundance of double-positive (CD4+/CD8+) T-cells that closely emulate normal thymocytes, along with B-cells with a mainly regulatory phenotype. The immature T-lymphocytes include many self-reactive clones, which manage to escape the altered and disorganized TME, without undergoing the physiological processes of positive and negative selection within the thymus parenchyma, serving as sources of autoimmune reactions. Thus, the plethora of immature double-positive (CD4+/CD8+) self-reactive T-cells renders the patients with these types of tumors more vulnerable to the emergence of autoimmune diseases, especially MG. The second immune signature, encountered in higher-grade thymic neoplasms, namely B3 and C classes, is characterized by fully differentiated cytotoxic T-cells, capable of eliciting de novo tumoricidal immune response. The generation of such an immunologically “hot” microenvironment, along with the higher expression levels of PDL-1 in such tumors, renders them more vulnerable to elimination by ICIs. Indeed, both completed and ongoing clinical trials have focused on the enrollment of patients with high-grade tumors for the evaluation of PDL-1 inhibitors. Preliminary results seem promising, with some kind of response or disease stabilization detected in a significant proportion of cases. However, as expected, this clinical effect is accompanied by a broad spectrum of corticosteroids-responsive immune-mediated adverse effects. 

It is clear that the accumulated knowledge regarding TET immune microenvironment has provided a deeper insight into the pathogenetic background of MG and has facilitated a better characterization and identification of predictive markers, concerning the effective implementation of ICIs, as a treatment approach for TET patients. However, a lot of questions still need to be answered, regarding the functional significance of different immune cell populations, in both autoimmunity predisposition and tumor-associated immune response. First of all, the exact functional specialization of innate immunity cellular subsets (dendritic cells, tumor-associated macrophages) has only been studied at a preliminary level. Moreover, B-cells, which represent the main exponent of humoral immunity, have not been thoroughly studied in the setting of tumor immune responses, including thymus malignancies, while their role in MG and other TET-related autoimmune conditions has only perfunctorily been studied. It is, therefore, important that future basic science research regarding thymic neoplasms steers towards these directions. A multi-dimensional approach, with a focus on the implementation of animal models that faithfully recapitulate human disease, is needed, so that these questions are pursued effectively.

Concerning clinical testing of immunotherapeutic regimens, further trials are required, preferably with larger patient cohorts, as the completed studies included small patient samples.

## Figures and Tables

**Table 1:** Ongoing clinical trials of ICIs on TETs.

Type of TET	Drugs	Number of Patients Enrolled	Trial Registry Number
TC, Thymoma	Avelumab	8	NCT03076554
TC, Thymoma	Nivolumab	NA	NCT03134118
TC, Thymoma	Pembrolizumab	NA	NCT03295227
TC, Thymoma	Pembrolizumab	40	NCT03858582
TC, Thymoma	Pembrolizumab/Sunitinib	NA	NCT03463460
TC	Pembrolizumab/Epacadostat	45	NCT02364076
TC, Thymoma	Pembrolizumab/Levatinib	43	NCT04710628
TC, Thymoma	Avelumab/Axitinib	38	CAVEATT
TC	Nivolumab/Vorolanib	117	NCT03583086
TC	Atezolizumab	34	NCT04321330
TC	KN046 (anti-PD-L1/CTLA-4 bispecific antibody)	66	NCT04469725
TC	Bintrafusp Alfa	38	NCT04417660
TC	Pembrolizumab/SO-C101 (IL-15/IL-15R α)	96	NCT04234113

## Data Availability

Not applicable.
